# Automatic Decision-Making Style Recognition Method Using Kinect Technology

**DOI:** 10.3389/fpsyg.2022.751914

**Published:** 2022-03-04

**Authors:** Yu Guo, Xiaoqian Liu, Xiaoyang Wang, Tingshao Zhu, Wei Zhan

**Affiliations:** ^1^Institute of Psychology, Chinese Academy of Sciences, Beijing, China; ^2^Department of Psychology, University of Chinese Academy of Sciences, Beijing, China; ^3^Information Science Research Institute, China Electronics Technology Group Corporation, Beijing, China

**Keywords:** Kinect, face data, machine learning, linear regression, decision-making style

## Abstract

In recent years, somatosensory interaction technology, represented by Microsoft’s Kinect hardware platform, has been widely used in various fields, such as entertainment, education, and medicine. Kinect technology can easily capture and record behavioral data, which provides new opportunities for behavioral and psychological correlation analysis research. In this paper, an automatic decision-style recognition method is proposed. Experiments involving 240 subjects were conducted to obtain face data and individual decision-making style score. The face data was obtained using the Kinect camera, and the decision-style score were obtained via a questionnaire. To realize automatic recognition of an individual decision-making style, machine learning was employed to establish the mapping relationship between the face data and a scaled evaluation of the decision-making style score. This study adopts a variety of classical machine learning algorithms, including Linear regression, Support vector machine regression, Ridge regression, and Bayesian ridge regression. The experimental results show that the linear regression model returns the best results. The correlation coefficient between the linear regression model evaluation results and the scale evaluation results was 0.6, which represents a medium and higher correlation. The results verify the feasibility of automatic decision-making style recognition method based on facial analysis.

## Introduction

Decision-making style is a habitual or unique behavior pattern that has an important influence on consumer behavior, career development, management, business, and other fields. To identify an individual’s decision-making style, researchers and companies generally use scales or questionnaires, such as Compensation Strategy Questionnaire (Zakay, 1990), General Decision-Making Style Questionnaire (GDMS) (Scott and Bruce, 1995), Decision-Making Inventory Questionnaire (Johnson, 1978), and Assessment of Career Decision-Making Inventory Questionnaire (ACDM) (Harren, 1979). Among them, the GDMS measures five dimensions of decision-making style, i.e., spontaneous, avoidant, rational, dependent, and intuitive (Scott and Bruce, 1995). The GDMS scale has been applied in different countries, populations, and age groups (Thunholm, 2008; Baiocco et al., 2009; Luke et al., 2012; Bavol’ar and Orosova, 2015; Delaney et al., 2015; Fischer et al., 2015; Alacreu-Crespo et al., 2019).

However, scale assessment is time-consuming and is not efficient enough in practical application scenarios such as enterprises. To address these issues, this research proposes a new method to measure decision-making style. Microsoft’s Kinect is a reliable 3D image capture hardware platform that has been widely used in multimedia interactions, such as games, education, and healthcare training. Recently, Kinect devices have has been widely used to support scientific research. With the latest version of the Kinect Software Development Kit (SDK) 2.0, the device can automatically isolate the face from the rest of the environment and use a grid structure of more than 1,000 3D coordinate points to represent the face (Zhang et al., 2016).

Machine learning involves algorithms that can learn and make predictions based on data. A model is constructed based on input (for example, in this paper, face data obtained by a Kinect) to manipulate such algorithms for data-driven prediction or decision making (Wei and Jia, 2017). Machine learning and data mining technology have achieved significant success in many engineering fields, including classification, regression, and clustering (Pan and Yang, 2010).

This study aims to explore the effective use of Kinect devices to recognize decision-making styles. Microsoft HD Face Basic SDK (Microsoft, 2014a) obtained a total of 1347 3D facial points. Then, 19200 time-frequency domain features were extracted. Next, principal component analysis (PCA) was used to reduce the dimensions from 19200 to 80. Finally, the GDMS score of subjects is used as machine learning annotation to establish a regression model. The predicted values of each dimension of the decision-making style obtained by the four algorithm models are compared with the actual values. The comparing results show that the linear regression model returns the best results.

The remainder of this paper is organized as follows. Section “Related Work,” which reviews studies related to decision-making styles, decision-making style recognition methods and psychological characteristics, and the use of Kinect to identify psychological characteristics. Section “Materials and Methods,” which introduces the proposed solution for decision-making style recognition using a Kinect device. Section “Results,” which introduces the experimental results and the evaluation of the effectiveness of our proposed solution. Issues related to the proposed solution and gender differences in decision-making styles and other behaviors are discussed in Section “Discussion,” and the conclusions of this study and future work are presented in the Section “Conclusion.”

## Related Work

### Decision-Making Styles

Decision-making style refers to the behavior pattern of an individual in a decision situation. Individual decision-making styles tend to be habitual, and people with different decision-making styles react differently in specific decision contexts based on established habits (Scott and Bruce, 1995). Decision-making style is an important factor affecting behavior in many fields, such as career development (Mau, 1995; Tinsley et al., 2002; Paivandy et al., 2008), team effectiveness (Verma et al., 2016), school choice (Ueichi et al., 2012), consumption (Zhu et al., 2012), traffic safety (Ju et al., 2019), medical care (Spalding and Edelstein, 2021), and gambling (Cosenza et al., 2019). At the same time, decision-making style is closely related to personality traits (Byrne et al., 2015; Wise et al., 2015; Iennaco et al., 2018; Farcic et al., 2020).

In order to measure the psychological characteristics of decision makers that affect influence the outcome of the decision, Scott and Bruce (1995) developed the General Decision Making Style questionnaire (GDMS) for decision making style recognition, an instrument of 25 items and five scales, provided evidence for five different decision-making styles: intuition (relying on hunches and feelings), rational (searching for and evaluating all alternatives), dependent (depending on other people’s suggestions), avoidant (delay or avoid making a decision), and spontaneous (characterized by immediacy and impulsivity). The compilation of the GDMS scale fully considered the control orientation (i.e., internal control orientation or external control orientation), occupational group, innovative behavior and innovativeness of the decision maker, and made up for the shortcomings of previous research. Scott and Bruce’s analysis of decision-making styles has been supported by subsequent research, including research on military officers’ decision-making (Thunholm, 2004), adolescent gambling decision-making (Cosenza et al., 2019), and research on the relationship between decision-making style and personality traits (Iennaco et al., 2018). In addition, it has been used to test the relationship between decision-making style and a series of decision-making performance results (Curşeu and Schruijer, 2012; Wood and Highhouse, 2014).

### Recognition Method of Decision-Making Style and Psychological Characteristics

Scale measurement is currently widely used methods to identify psychological characteristics, which is rigorous, accurate, and reliable. In addition, the scale has established a good norm, and the measurement results can be directly compared with the norm. However, scale measurement also has limitations that cannot be ignored. For example, the correct use of scales requires that the main tester have certain psychological knowledge, understand the content of the scale, and be familiar with the skills of using the scale. In addition, education level, cultural differences, differences in a physical or emotional state when filling the scale, similar exercises, and experience will affect the results of the scale (Bai et al., 2013; Li et al., 2019). Therefore, a more efficient method of measuring decision-making styles that can eliminate the interference of human factors is required.

In recent years, there has been a new trend in the measurement of decision-making style that focuses on characteristics, e.g., gait, posture, and expression, rather than traditional scales. For example, Connors et al. (2013) proposed to predict differences in individual decision-making styles by analyzing signatures and gestures based on motion pattern analysis, which is an observation method that can objectively encode specific body movements to provide an indicator of decision-making style. In addition, Xue and Wang (2012) used the self-developed facial awareness scale and consumer style scale to find a significant correlation between facial awareness and consumer decision-making characteristics.

Machine learning technologies are widely used in various fields of psychology research; thus, they play important functions in data processing, experimental control, and psychological feature recognition. For example, Yacoob and Davis (1994) proposed a facial dynamics analysis and representation method to recognize facial expressions from image sequences to identify emotions based on static images. Bai et al. (2013) used Weibo data to predict users’ Big Five personality traits. In addition, Heraz and Clynes (2018) identified subjects’ emotions by measuring the coordinates, strength, and skin area of finger touch screens while operating their phones, and Chen et al. (2019) proposed a method to identify decision-making styles using text, images, and videos (digital footprints) on Facebook. Siddiqui et al. (2021) developed a wearable sensor-based platform to identify autism spectrum disorder (ASD) using machine learning. Asaduzzaman et al. (2021) developed a system by using machine learning and data mining approach to predict the risk level of cervical and ovarian cancer in association to stress. Existing research has proved that the machine learning model has achieved a performance that is not inferior to the traditional scale measurement. Gonzalez (2020b) considered using machine learning technology to reduce the length of the scale and improve the efficiency of scale measurement. Oh et al. (2017) trained an artificial neural network classifier to classify suicide attempts by high-risk groups and evaluated the classifier. The results show that this classifier based on 31 psychiatric scales and 10 sociodemographic elements can be trained predict suicide attempts accurately. Mazza et al. (2019) used machine learning methods to detect fake personalities in male samples. The results show that machine learning technology has achieved higher performance than traditional psychometric techniques in detecting counterfeiters in self-reported personality questionnaires.

### Using Kinect to Identify Psychological Characteristics

The development of video capture tools has realized different ways to measure decision-making styles. Previously, video capture was achieved using images acquired by traditional cameras. With the development of depth sensors, particularly with the emergence of the Microsoft Kinect, an increasing number of algorithms use depth data in vision-based human action recognition. The Kinect conveniently provides high-resolution, real-time depth information (Chen et al., 2013; Bonnechère et al., 2014). In previous studies, Zhang et al. (2016) proposed a new method of emotion recognition via facial expression changes captured by Kinect sensor over a period of time, Han et al. (2016) presented a new facial expression feature based on relevant distances from salient points on a face mesh to a set of reference points collected from the new version of the Microsoft Kinect device, and a facial expression similarity measure based on the principle of dynamic time warping, Boutellaa et al. (2015) explores the usefulness of the depth images provided by the current Microsoft Kinect sensor in different face analysis tasks including identity, gender and ethnicity, Silverstein and Snyder (2017) presented a straightforward implementation of facial recognition using the Microsoft Kinect sensor for the purpose of patient identification in a radiotherapy setting.

## Materials and Methods

### Motion Capture Device

We used The Microsoft Kinect to capture subjects’ facial movements. The Kinect for Windows software development kit (SDK 2.0) is the latest version of the program development kit used to develop applications that support Kinect.

### Subjects

We recruited 240 subjects (110 males and 130 females) age 18–39 years (*M* = 22.83, *SD* = 2.79). All subjects had education levels ranging from high school to graduate school, and were in good health and had no disease affecting the face and facial expressions. Table 1 show the demographic information of the subjects. Note that this experiment was approved by the Scientific Research Ethics Committee of the Institute of Psychology, Chinese Academy of Sciences (Approval No.: H15010).

**TABLE 1:** Demographic information of subjects.

Demographic Information	*n*	*%*
**Gender**		
Female	130	54.20
Male	110	45.80
**Marital status**		
Single	138	57.50
Partnered	96	40.00
Married	4	1.67
Divorced	2	0.83
**Educational background**		
High school	2	0.83
In college	35	14.60
University/higher vocational	36	15.00
In graduate school	152	63.30
Postgraduate	15	6.25
**Household income^a^**		
Unknown	9	3.75
<1	11	4.58
1–5	50	20.80
5–10	84	35.00
10–30	76	31.67
30–60	8	3.33
>60	2	0.83

*N = 240. Subjects were on average 39.5 years old (SD = 10.1), and subjects age did not differ by condition.*

*^a^The unit of family income is ten thousand yuan RMB.*

### Data Collection

In this experiment, the main tester first introduced the purpose, process, and privacy protection information to the subjects. The subjects agreed to participate in the experiment and provided signed informed consent.

The subjects were numbered to facilitate the accurate matching of the test scale evaluation results with the corresponding facial activity data in the data collection process.

The subjects were given a speech outline (refer to the Appendix for additional information). Each subject prepared for 5 min and then completed not less than 2 min of self-introduction according to the speech outline. Then, each subject filled in the Chinese version of the GDMS to measure the five dimensions of decision-making style.

### Data Processing

**FIGURE 1:**
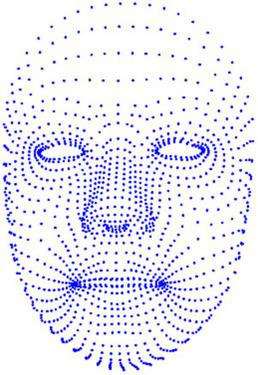
A Kinect face image with 1347 facial recognition points.

The Kinect can detect the 3D coordinates of 1347 points on the face, and a total of 1347 × 3 = 4041 time-series data can be obtained at a sampling frequency of 30 Hz. Figure 1 show the distribution of points on the face.

#### Random Stratified Sampling Distribution of General Decision-Making Style Questionnaire Results

The method of establishing the machine learning model in this study is supervised learning. Therefore, the GDMS score data set needs to be divided into a training set and a validation set. According to the GDMS score, the samples were randomly stratified sampling distribution. Divided the GDMS score from high to low into four areas, each with 60 samples. When selecting the training set and the validation set, according to the ratio of 8:2, 80% of the samples in each area were selected as the training set, and the remaining 20% of the samples were selected as the validation set, that is, 48 samples in each area were selected as in the training set, 12 samples were selected as the validation set. Totally, 192 samples were selected as the training set, and 48 samples were selected. This ensured that each fraction was distributed evenly in the training and verification sets, avoided the large difference in score between the training set and the validation set, resulting in poor performance of the trained model.

#### Kinect Coordinate System

Kinect use 3D coordinate system. The coordinate system is defined as follows:

The origin (x = 0, y = 0, z = 0) is located at the center of the IR sensor on Kinect;
X grows to the sensor’s left;
Y grows up (note that this direction is based on the sensor’s tilt);
Z grows out in the direction the sensor is facing;
1 unit = 1 meter.

#### Coordinate Shift

The origin of the coordinate system is the camera, and the height and weight of each subject differed; thus, the coordinates of each sample differed and could not be compared. Thus, the coordinate system must be unified. Here, we took the vertex of each face as the origin and then subtracted the coordinates of the remaining points from the coordinates of the vertex. For example, if the vertex coordinates are x1, y1, z1, and the coordinates of the second point are x2, y2, z2, we obtain the coordinates of the new point as follows:

x12 = x2 – x1,
y12 = y2 – y1,
z12 = z2 – z1.

#### Mean Filtering

During the subject’s speech, the slight vibration of the facial muscles, the noise generated by the Kinect sensor itself, the slight vibration of the ground, and the change of the ambient light will all cause noise in the facial data collected by the Kinect. So mean filtering is used to remove this noise. Mean filtering uses each data point within a window containing a certain number of data points (*W* = 3 or *W* = 5, in this paper) to calculate the arithmetic mean, instead of the value of the central data in the window. This eliminates the noise data in the face data.

Here, three or five adjacent frames of each coordinate are averaged. For example, if the three coordinates of a point are x, y, z, the value of each coordinate after mean filtering is calculated as follows, n represents the number of adjacent frames.


(1) $$\text{P}\left(x,y,z\right)=\left\{ \begin{array}{c} \frac{1}{n}\sum_{i}^{i+n}x_{i}\\ \frac{1}{n}\sum_{i}^{i+n}{\text{y}}_{i}\\ \frac{1}{n}\sum_{i}^{i+n}{\text{z}}_{i} \end{array}\right.\;n=3,5$$

#### Data Filtering

Time is required for the subjects to enter the state at the beginning of the self-introduction and close the Kinect at the end of the speech; thus, the first and last 5 s (i.e., the first and last 150 frames) were removed from each sample. Then, to facilitate using a sliding window to take the feature value and perform fast Fourier transform (FFT) on an array of each window, the number of frames of each sample is retained 66*32 = 2112. Among the 240 face data samples, there are four samples which the number of frames is less than 2112 frames after the above processing, and these samples are discarded. In this way, we obtained 236 samples finally.

#### Screening Facial Points With the Largest Rate of Change

During the subject’s speech, the Kinect device collected data of 1,347 facial points. The changes of each facial point were very subtle, and the Kinect SDK did not mark the position of each point. To optimize the performance of the machine learning model, we selected the top 100 facial points with the largest Euclidean distance variation for modeling. In low dimensions, the Euclidean distance plays a significant role, so it is widely used in the field of image processing.

Each frame of the sample included 1347 facial points, and the Euclidean distance of each face point adjacent frame was calculated. The difference of the j-th face point in the i-th frame was calculated using Equation (2), where i∈{1,…, N – 1}, j∈{1,…,1347}. Thus, we obtained the (N – 1) × 1347 matrix D.


(2) $$d_{i,j}=\sqrt{\sum_{i=0}^{N-1}{\left(p_{i+1,j\_m}-p_{i,j\_m}\right)}^{2}}\;m\in \left\{x,y,z\right\}$$

Then, the variance of each column in the matrix was calculated as follows.


(3) $$\begin{array}{c} \sigma_{\text{j}}=\frac{1}{N-1}\sum_{i=0}^{N-1}{\left(d_{i,j}-\bar{d_{j}}\right)}^{2}\;i=0,1,2,\ldots ,N-1\\ \;j\in \left\{0,1,2,\ldots ,1346\right\} \end{array}$$

Among the above equation, i is the row number of the matrix, and j is the column number. The calculation result was stored in a matrix, where each row represents the variance of the Euclidean distance of all adjacent points of a sample. This matrix is expressed as follows.


(4) $$\begin{array}{c} Dv=\left(\begin{array}{cccc} v_{1,1} & v_{1,2} & \cdots & v_{1,1347}\\ v_{2,1} & v_{2,2} & \cdots & v_{2,1347}\\ \vdots & \vdots & \vdots & \vdots \\ v_{236,1} & v_{236,2} & \cdots & v_{236,1347} \end{array}\right) \end{array}$$

Then, calculate the average variance of each column of the matrix Dv and sort them in descending order, so that the facial points are sorted according to the rate of change from high to low. Take the first 100 points.

#### Feature Extraction

Before establishing the decision-making style model, feature extraction of face data is needed to obtain useful information as the feature value. Here, FFT is used to extract temporal-frequency feature of face data. The FFT is a wavelet transform method to extract information of time domain-spectra into the frequency-domain spectra in order to obtain both frequency and temporal information on signals and have been used in digital signal processing applications (Duhamel and Vetterli, 1990).

Features were extracted for each face point separately. Here, a sliding window was used to fetch the features. Sliding window is used to perform required operation on specific window size of given array.

Here, for each axis, each 128 frames is a FFT window, and the value of each frame is the coefficient of FFT.


(5) $$F_{k}=\sum_{\text{n}=0}^{N-1}x_{n}e^{-i2\pi k\frac{n}{N}}\;k=0,1,\ldots \ldots N-1$$

Among the above equation, N represents the number of frames of face data, and i is a complex number. Calculation result _*F_k_*_ consists of real and imaginary parts.

After each calculating FFT, the window slides 64 frames, that is, the window coverage rate is 50%.

Then, the modulus of each coefficient of the FFT result was calculated as follows.


(6) $$\left|Z\right|=\sqrt{X^{2}+Y^{2}}$$

Among the above equation, X and Y are the real and imaginary parts of the complex number. As a result, 128 modules were obtained. The variance [Equation (2)] and average of these 128 modules are calculated, which are taken as the two feature values. Then, the window slides 64 frames, and the same operation is repeated. As described in section “Data Filtering”, each face data sample has 2112 frames, so the sliding window will get 32 sequences. So the number of feature values for each axis is: 32 × 2 = 64. Each face data has x, y, and z axes, so the number of feature values extracted from each facial data sample is 32 × 2 × 3 = 192. Therefore, the number of feature values extracted from each face data sample is: 192 × 100 = 19200.

#### Feature Dimensionality Reduction

Recently, large data sets have become increasingly common, and such large amounts of data are frequently difficult to interpret. PCA is used to reduce the dimensionality of such datasets and increase interpretability while minimizing information loss (Jolliffe and Cadima, 2016).

As mentioned above, in this study, the number of eigenvalues extracted was 19200, and the number of frames of each sample was 2112. As the number of eigenvalues is much greater than the number of frames, overfitting is likely, which would result in many errors in the model’s validation test set. Thus, dimension reduction was required. Here, after dimensionality reduction, the number of eigenvalues was reduced from 19200 to 80, which included more than 98% variance contribution of all eigenvalues (before dimensionality reduction).

### Model Training

The method of establishing machine learning models in this study is supervised learning. The machine learning model is trained based on the face data in the training set and the GDMS score, and then the model calculates the GDMS score based on the face data in the validation set, and compares it with the actual GDMS score in the validation set to get the correlation coefficient between the two. In this study, we used a python-based open source framework to train four machine learning models and evaluated their performance.

## Results

As described in Section “Mean Filtering”, when mean filtering is performed, three or five adjacent frames of each coordinate are averaged. Therefore, *W* = 3 and *W* = 5 are used here to represent the results of data processing and modeling using two methods of mean filtering.

### General Decision-Making Style Questionnaire Scale Analysis

The average score and standard deviations of the five dimensions of the GDMS scale filled by the subjects are shown in Table 2. As can be seen, the score for males is significantly greater than that for women for the perception, rationality, and impulse dimensions, and the score for men are significantly lower than those of women for the dependence and avoidance dimensions.

**TABLE 2:** Average score of GDMS scale in five dimensions.

Gender	Spontaneous		Avoidant		Rational		Dependent		Intuition	
	*M*	*SD*	*M*	*SD*	*M*	*SD*	*M*	*SD*	*M*	*SD*
Female	17.67	4.60	18.06	2.61	16.71	4.33	15.21	4.02	18.49	3.53
Male	15.66	4.52	19.90	3.13	15.20	4.12	17.00	4.86	17.05	3.62

### Algorithm Model

Four algorithms were used to establish the decision-making style model, i.e., the Linear Regression (LR), Bayesian Ridge Regression (BR), Support Vector Regression (SVR) with a linear kernel function, and built-in cross-validation Ridge regression (RidgeCV). By default, RidgeCV performs general cross-validation, which is an effective form of leave-one-out cross-validation. Then, 10-fold cross-validation was employed to verify the effect of the model.

### Validity of Algorithm Model

Table 3 compare the Pearson correlation coefficient between the predicted value and the actual value calculated by each algorithm for *W* = 3 and *W* = 5.

**TABLE 3:** Correlation coefficient between the predicted and actual values of decision-making style of the four algorithms models.

Algorithms	Spontaneous	Avoidant	Rational	Dependent	Intuition	*M* ^a^	*SD* ^b^
**LR**							
*W* = 3	0.60**	0.44*	0.45*	0.57**	0.40*	0.49	0.078
*W* = 5	0.45*	0.44	0.50**	0.61**	0.41	0.48	0.070
**SVR**							
*W* = 3	0.41	0.27	0.27	0.41*	0.39*	0.35	0.066
*W* = 5	0.38	0.27	0.28	0.43*	0.38	0.35	0.062
**RidgeCV**							
*W* = 3	0.48**	0.37	0.45**	0.52**	0.40*	0.44	0.054
*W* = 5	0.45*	0.41*	0.44**	0.53**	0.43*	0.45	0.041
**BR**							
*W* = 3	0.42**	0.34*	0.48**	0.53**	0.38*	0.43	0.068
*W* = 5	0.42*	0.41*	0.51**	0.52**	0.33*	0.44	0.070
***M* ^c^**							
*W* = 3	0.48	0.36	0.41	0.50	0.39		
*W* = 5	0.43	0.38	0.43	0.52	0.39		
***SD* ^d^**							
*W* = 3	0.076	0.061	0.083	0.059	0.008		
*W* = 5	0.027	0.044	0.081	0.049	0.007		

*In the column 1, W refer to the size of the sliding window used to eliminate noise.*

*^a^W = 3 and W = 5 for each algorithms, the means value of correlation.*

*^b^W = 3 and W = 5 for each algorithms, the standard deviation value of correlation.*

*^c^W = 3 and W = 5 for each GDMS dimension, the means value of correlation.*

*^d^W = 3 and W = 5 for each GDMS dimension, the standard deviationvalue of correlation.*

*p-value < 0.005.

**p-value < 0.001.

### Comparison of Recognition Effect of Different Algorithm Models

With the Bayesian ridge regression algorithm model, the correlation coefficient of the avoidant dimension was less than 0.4 for *W* = 3; however, the others were greater than 0.4. With the linear regression algorithm, the correlation coefficient of the avoidance and intuitive dimensions was less than 0.4 for *W* = 5, and the others were greater than 0.4. For the RidgeCV algorithm, the correlation coefficient of the spontaneous dimension was close to 0.6 for *W* = 3, and the correlation coefficient of the avoidant dimension is less than 0.4. In addition, for *W* = 5, the correlation coefficients of the five dimensions were all greater than 0.4. With the support vector regression algorithm model, the avoidant dimension of *W* = 3 and the intuitive of *W* = 5. The correlation coefficient of the dimension is less than 0.4, and the rest are more than 0.4.

For the model established using the BR algorithm, the average correlation of the five dimensions was 0.454 (for *W* = 3), and the standard deviation was 0.071. For the model established using the linear regression algorithm, the average correlation of the five dimensions was 0.471, and the standard deviation was 0.068. For the model established using the RidgeCV algorithm, the average correlation of the five dimensions was 0.478, and the standard deviation was 0.085. For the model established using the support vector regression algorithm model, the average correlation of the five dimensions was 0.348, and the standard deviation was 0.064. When *W* = 3 and *W* = 5, there is no significant difference in the recognition effect of the four models.

### Comparison of Model Recognition Effect for *W* = 3 and *W* = 5

Table 3 show that all four algorithm models, no matter *W* = 3 or *W* = 5, have the best effect in identifying the dependent dimension of GDMS, and the correlation is above 0.4. Among them, the correlation of the linear regression model to identify the rational dimension reached 0.61.

### Recognition Performance of Models Across Dimensions of Decision-Making Style

Table 2 shows that, regardless of male or female, the standard deviation of scores on the Avoidant dimension of the GDMS is significantly lower than the other four dimensions. The other four dimensions are Intuition, Rational, Dependent, and Spontaneous in ascending order from low to high according to standard deviation. Table 3 shows that the mean values of the correlation coefficients between the predicted value and the actual value of the five dimensions of decision-making style calculated by the four algorithm models are, in descending order, Avoidant, Intuition, Rational, Spontaneous, and Dependent. This shows that the performance of the algorithm model is significantly related to the discreteness of the GDMS scores. If all subjects have a small difference in scores on a dimension, the model will perform poorly on that dimension as well.

### Root Mean Square Error of Algorithm Model

Root mean square error (RMSE) is one of the criteria for evaluating the performance of machine learning models. The RMSE of the predicted value is the square root of the average of the squared error of the measurement, that is, the square root of the mean square error between the predicted value and the actual value. The calculation process of RMSE is similar to the standard deviation, but their research objects and research purposes are different. Standard deviation is used to measure the degree of dispersion of a set of numbers, and RMSE is used to measure the deviation between the predicted value and the actual value.

Table 4 shows the RMSE of the predicted and actual values calculated by the four algorithm models when *W* = 3 and *W* = 5. All algorithms have minimum RMSE in dependent dimensions.

**TABLE 4:** Root mean square error of the four algorithms.

Algorithms	Spontaneous	Avoidant	Rational	Dependent	Intuition
**LR**					
*W* = 3	4.23	5.14	4.73	3.16	4.58
*W* = 5	3.67	5.21	4.53	3.02	4.81
**SVR**					
*W* = 3	3.54	5.43	4.38	3.35	5.36
*W* = 5	3.58	5.6	4.44	3.29	5.49
**RidgeCV**					
*W* = 3	3.50	5.34	3.92	3.17	5.13
*W* = 5	3.53	5.33	3.90	2.87	5.11
**BR**					
*W* = 3	3.48	5.33	4.60	3.20	5.35
*W* = 5	3.50	5.20	4.60	3.11	5.35

### Split-Half Reliability of Algorithm Model

Split-half reliability estimates are common indicators of internal consistency reliability, which is applicable whenever a measurement consists of repeatedly administered trials (Steinke et al., 2021).

Windows of two lengths (i.e., *W* = 3 and *W* = 5) were used for mean filtering, modeled separately. Divide all samples into two halves according to odd and even frames, and input the algorithm model generated before. We calculate the split-half reliability of the models established by these two mean filtering methods for comparison of internal consistency, as shown in Table 5. The results showed that the split-half reliability of the two methods was not significantly different.

**TABLE 5:** Split-half reliability of the four algorithms.

Algorithms	Spontaneous	Avoidant	Rational	Dependent	Intuition
**LR**					
*W* = 3	0.70**	0.73**	0.70**	0.76**	0.69**
*W* = 5	0.68**	0.77**	0.73**	0.71**	0.68*
**SVR**					
*W* = 3	0.41**	0.24	0.49*	0.20	0.23*
*W* = 5	0.47*	0.33	0.47	0.51	0.20*
**RidgeCV**					
*W* = 3	0.63**	0.39*	0.76**	0.39**	0.42**
*W* = 5	0.61**	0.45*	0.72**	0.43*	0.52**
**BR**					
*W* = 3	0.54**	0.69*	0.69*	0.49**	0.59
*W* = 5	0.53*	0.62*	0.71**	0.53*	0.61

*In column 1, W refer to the size of the sliding window used to eliminate noise.*

*p-value < 0.005.

**p-value < 0.001.

## Discussion

### Gender Differences in Decision-Making Styles and Other Behavioral Performance

In this study, The correlation between the score of the decision-making style predicted by the machine learning model and the actual score of GDMS does not show a significant difference in gender. However, Table 2 shows that significant gender differences were observed in the score of the five dimensions of the GDMS scale. Existing studies support this result. Alacreu-Crespo et al. (2019) show that men score higher on three dimensions, i.e., impulse, intuition, and rationality. Delaney et al. (2015) show that women score higher on the dependency dimension and men score higher on the impulse dimension. Salo and Allwood (2011) research that focuses on police officers shows that the decision-making style of female police officers is biased toward low rationality, high dependence, and high avoidance.

Other differences between genders have been documented. In terms of attitudes toward the new crown epidemic, women are more likely than men to regard the new crown epidemic as a serious health problem. Thus, women are more willing to comply with prevention and control policies (Galasso et al., 2020). When dealing with individuals in a lonely environment, women are more likely to perform prosocial behaviors, while men are less cooperative and less likely to perform prosocial behaviors (Huang et al., 2016), which supports women’s higher score on the dependency dimension of the GDMS. Denneson et al. (2020) studied the gender differences in suicide risk among U.S. military veterans and found that women are more likely to be affected by negative relationships, leading to increased suicide risk. In contrast, men will not seek external emotional support, which may lead to suicidal behavior because they do not have a clear life goal. In a study on gambling behavior, Carneiro et al. (2020) found that men participate in gambling at a younger age than women, and that men’s risk of gambling is 2.3 times higher, and the risk of gambling-related problems is 3.6 times higher. In terms of risk decision-making, studies have shown that men’s risk preference is higher than women’s (Byrnes et al., 1999). On the contrary, women are more inclined to avoid risks than men (Powell and Ansic, 1997).

### Relationships Between Facial Expressions and Decision-Making Style

A study has shown that the amygdala, which has long been thought to be associated with emotion and alertness, is associated with decision-making and social behavior, indicating that facial expressions and decision-making styles are biologically related to a certain extent (Chang et al., 2015). de Melo et al. (2014) proposed an inference mechanism that retrieves information about others’ evaluations from emotional expressions and then infers the mental states of others. However, Melo’s research also shows that the same expression can be interpreted as different information in different contexts. It can be inferred that in this study, if the subjects are in different situations, the performance of the established decision-making style algorithm model will be affected. We asked all subjects to answer the same questions, eliminating irrelevant variables as much as possible.

### Prediction Effects of the Four Algorithm Models

In this study, the predicted values of each dimension of the decision-making style obtained by the four algorithm models are compared with the actual values. The comparison indicates a certain degree of correlation between that the predicted and actual values. However, the recognition effect of each model is different. The experimental results show that the linear regression model achieved the best results. The correlation between the predicted value of the five dimensions of the linear regression model is the hightest in the four algorithm models, and the standard deviation of the linear regression model is low in the four algorithm models (Table 3). When the sample size is small, the other three algorithms are prone to overfitting. Although the accuracy of the linear regression model is also affected to a certain extent (Overfitting, as mentioned above), the performance of linear regression is still slightly higher than the other three algorithms when the sample is small. Related research also shows that the linear regression algorithm and its improved algorithm show prediction accuracy comparable to random forest, support vector machine, and the random generalized linear model (Cardoso-Silva et al., 2019; Rath et al., 2020; Meulenbroek and Pichardo, 2021; Nguyen et al., 2021).

The RMSE is also an important criterion for evaluating regression model. In this study, there is no significant difference in RMSE of all algorithms. In existing researches on identifying psychological characteristics, physiological characteristics or mental illness, the RMSE performance of various algorithm models is different, no one has obvious advantages (Memarian et al., 2017; Haun et al., 2021; Zhang et al., 2021). This may be caused by different data collection and modeling method in each research.

In this study, the split-half reliability of the linear regression model is higher than that of other algorithm models, reflecting higher internal consistency. One previous study used a linear regression algorithm to establish a model to distinguish multi-dimensional psychological symptoms. Split-half reliability was one of the methods to evaluate the model and achieved a moderate or above correlation (Wang et al., 2020).

### Key Facial Points

**FIGURE 2:**
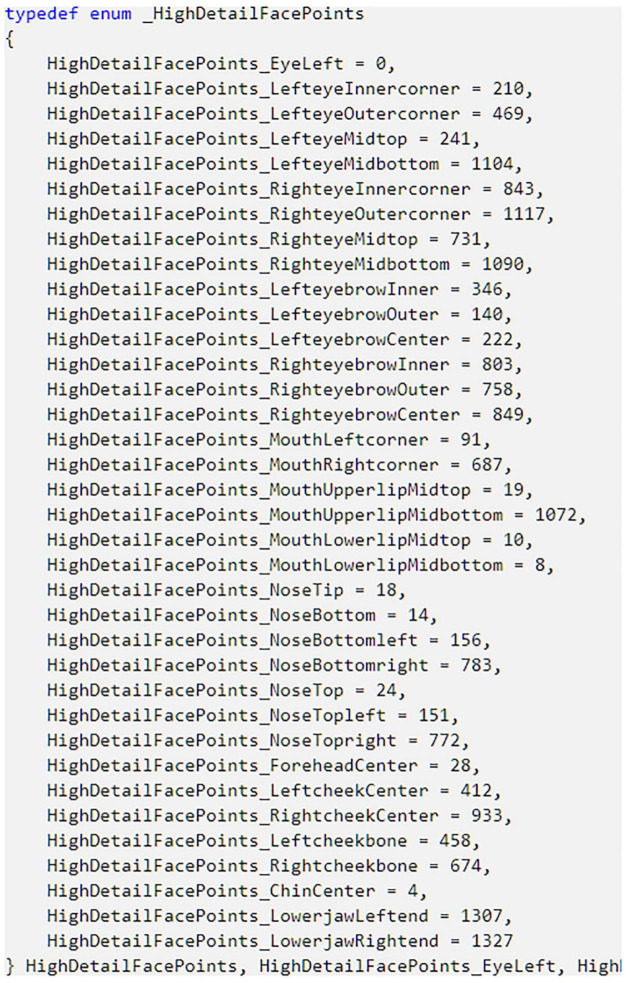
The Specific information of 36 key facial points. In the enumeration type, for each element, the variable name on the left side of the equal sign represents the position of the key point on the face, and the value on the right side of the equal sign represents the ID of this point among all 1347 facial points.

In this study, all 1347 facial points were used for modeling. Since the rate of change of each point is different, some non-key points have little effect on the establishment of the model. Although it is impossible to identify the specific location of each facial point, Kinect SDK provides 36 key facial points, which represent 36 parts of the face (Microsoft, 2014b). Figure 2 show the specific information of 36 key facial points. Han et al. (2016) used key facial points for face recognition and achieved good results. Based on these key points, the changes of several points in the nearby area can be inferred, so that the changes of facial points located in important areas such as eyes, lips, nose, etc. can be obtained.

### Setting of Questions in the Speech Outline

In order to collect face data, we set up three questions in the speech outline. These three questions are all neutral, tried to avoid the subject’s emotional changes. In the future work, we will record the three questions separately, and add a questionnaire after each question to test the emotions at that time to ensure that all the facial expressions are in the expected emotional state. When each subject is asked to answer each question, the expression time is not less than 2 min.

Most of our subjects are undergraduates and graduate students, so the questions are also targeted. Considering expanding the scope of the sample and the expression of different emotions, the three questions can be modified to introduce yourself (neutral), the happiest thing recently (positive), and tell a sad thing you experienced personally (negative).

### Other Analysis Methods

The decision-making style recognition method proposed in this research does not involve the classification of psychological characteristics. In fact, machine learning is widely used for classification. Existing research collected physiological characteristics (Darzi et al., 2019; Azari et al., 2020), behavioral performance (Darzi et al., 2019; Leiding et al., 2021), personality characteristics (Darzi et al., 2019; Azari et al., 2020), electronic medical records (Edgcomb et al., 2021) as training data training models, and use the established model to classify indicators such as emotions (Azari et al., 2020), behavioral disorders (Gonzalez, 2020a), depression (Edgcomb et al., 2021) and violent crime risk (Leiding et al., 2021), and achieved valuable research results. In addition, the model established in this study is a supervised learning that uses labeled data for training. Some studies compare the training methods of supervised classification and unsupervised classification (Azari et al., 2020), which has practical significance.

### Advantages of the Proposed Method

Compared with the traditional scale-based method of identifying decision-making styles, the proposed method has the following advantages: first, it has high measurement efficiency and is convenient for large-scale group measurements. Second, because the Kinect3D device is used to collect the subject’s facial point data, there is no main tester to participate, which avoids the main tester’s interference with the subjects. Third, the test scene is simple and can be adapted to various locations. In summary, the proposed method is simple to implement, inexpensive, and can be applied to scientific research and enterprises.

### Limitations

This study has the following limitations. First, if we want to improve the correlation between the model and the GDMS scale results and improve the recognition accuracy, the diversity of the subjects, should be enriched to include a wider range of educational backgrounds and ages. Since the scope of sample acquisition can only be mainly based on college students and graduate students, there may be a certain degree of bias. We will consider improving the sample composition. Second, the Kinect3D device has been discontinued, and the Kinect SDK is no longer updated. Therefore, it is necessary to explore alternatives, such as more common camera equipment and open source facial recognition framework, and optimize our algorithm model to improve the performance of decision-making style recognition.

## Conclusion

The existing decision-making style recognition methods are mainly based on scale evaluation. However, the scale evaluation is time-consuming and not suitable for large-scale measurement. This research proposes a method to recognize decision-making styles automatically. The proposed method is based on subjects’ facial points collected by a Kinect3D device. The results indicate that the proposed method is a practical approach to decision-making style recognition. Using somatosensory equipment represented by Kinect combined with machine learning technology to identify decision-making styles or other psychological characteristics is a feasible research direction. At the current stage, the method proposed in this study can be used as an effective aid for scale measurement. In the future work, if this study is applied to the actual scene, it is necessary to seek for the more common camera equipment, open source facial recognition framework and improved algorithms.

## Data Availability Statement

The raw data supporting the conclusions of this article will be made available by the authors, without undue reservation.

## Ethics Statement

The studies involving human participants were reviewed and approved by Scientific Research Ethics Committee of the Institute of Psychology, Chinese Academy of Sciences. The patients/participants provided their written informed consent to participate in this study.

## Author Contributions

TZ contributed to the conception and design of the study. XL collected the data, developed the instrument, and provided guidance for data preprocessing and model establishment. WZ guided the experimental design and provided comments on data processing methods. XW provided guidance for the reliability and validity testing plan. YG performed the statistical analysis, trained the decision-making style models, and wrote the manuscript with input from all authors. All authors contributed to the article and approved the submitted version.

## Conflict of Interest

WZ was employed by the company China Electronics Technology Group Corporation. The remaining authors declare that the research was conducted in the absence of any commercial or financial relationships that could be construed as a potential conflict of interest.

## Publisher’s Note

All claims expressed in this article are solely those of the authors and do not necessarily represent those of their affiliated organizations, or those of the publisher, the editors and the reviewers. Any product that may be evaluated in this article, or claim that may be made by its manufacturer, is not guaranteed or endorsed by the publisher.

## Appendix | Behavioral Data Collection Process (Lecture)

### (1) Process

(1.1) Register and number subjects.

(1.2) Give each subject a speech outline for self-introduction and provide 5 min for preparation, and complete at least 2 min of self-introduction.

(1.3) Before the speech, confirm that the Kinect can correctly capture facial movements, and confirm that the serial number of the subjects’ Kinect data file is consistent with the serial number when the subjects fill in the GDMS scale.

(1.4) The subjects began to speak and the Kinect records the subjects’ facial movements.

(1.5) End of speech.

### (2) Speech Outline:

(2.1) Please introduce yourself and describe your hometown in detail.

(2.2) Please describe your major and your research work during your studies in detail.

(2.3) Please outline your plans for the future and what type of work you want to engage in.
